# Dicyclo­hexyl­{3-hydr­oxy-*N*′-[1-(2-oxidophenyl-κ*O*)ethyl­idene]-2-naphthohydrazidato-κ^2^ *N*′,*O*}tin(IV)

**DOI:** 10.1107/S1600536810005829

**Published:** 2010-02-17

**Authors:** See Mun Lee, Hapipah Mohd Ali, Kong Mun Lo, Seik Weng Ng

**Affiliations:** a Department of Chemistry, University of Malaya, 50603 Kuala Lumpur, Malaysia

## Abstract

In the title compound, [Sn(C_6_H_11_)_2_(C_19_H_14_N_2_O_3_)], the Sn^IV^ atom is *O*,*N*,*O*′ chelated by the deprotonated Schiff base ligand and exists in a *cis*-trigonal-bipyramidal environment, completed by the two cyclohexyl ligands.

## Related literature

For other dialkyl­tin(IV) compounds with similar Schiff-base ligands, see: Lee *et al.* (2009*a* ▶,*b* ▶,*c* ▶).
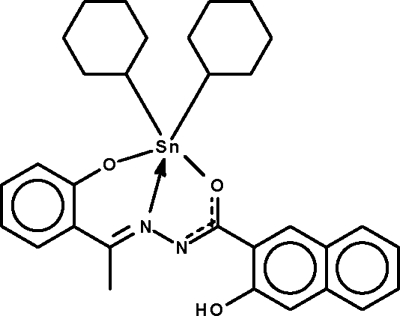


## Experimental

### 

#### Crystal data

[Sn(C_6_H_11_)_2_(C_19_H_14_N_2_O_3_)]
*M* *_r_* = 603.31
Monoclinic, 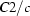

*a* = 30.2358 (4) Å
*b* = 7.7030 (1) Å
*c* = 25.8528 (4) Å
β = 111.249 (2)°
*V* = 5611.92 (16) Å^3^
*Z* = 8
Mo *K*α radiation
μ = 0.95 mm^−1^
*T* = 293 K
0.30 × 0.10 × 0.10 mm

#### Data collection

Bruker SMART APEX diffractometer
Absorption correction: multi-scan (*SADABS*; Sheldrick, 1996 ▶) *T*_min_ = 0.765, *T*_max_ = 0.912
26097 measured reflections
6424 independent reflections
3942 reflections with *I* > 2σ(*I*)
*R*_int_ = 0.047

#### Refinement

*R*[*F* ^2^ > 2σ(*F* ^2^)] = 0.043
*wR*(*F* ^2^) = 0.111
*S* = 1.00
6424 reflections
336 parameters
H-atom parameters constrained
Δρ_max_ = 0.52 e Å^−3^
Δρ_min_ = −0.43 e Å^−3^

### 

Data collection: *APEX2* (Bruker, 2009 ▶); cell refinement: *SAINT* (Bruker, 2009 ▶); data reduction: *SAINT*; program(s) used to solve structure: *SHELXS97* (Sheldrick, 2008 ▶); program(s) used to refine structure: *SHELXL97* (Sheldrick, 2008 ▶); molecular graphics: *X-SEED* (Barbour, 2001 ▶); software used to prepare material for publication: *publCIF* (Westrip, 2010 ▶).

## Supplementary Material

Crystal structure: contains datablocks global, I. DOI: 10.1107/S1600536810005829/ci5035sup1.cif

Structure factors: contains datablocks I. DOI: 10.1107/S1600536810005829/ci5035Isup2.hkl

Additional supplementary materials:  crystallographic information; 3D view; checkCIF report

## Figures and Tables

**Fig. 1.**
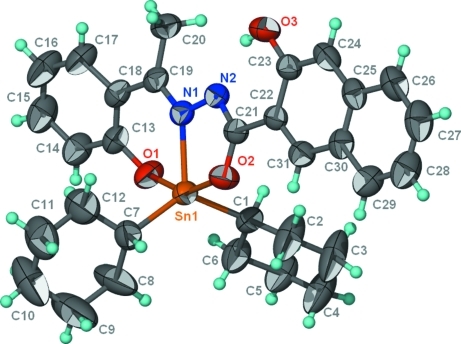
Displacement ellipsoid plot (Barbour, 2001) of [Sn(C6H11)2(C9H14N2O3)] at the 50% probability level. H atoms are drawn as spheres of arbitrary radius.

## supplementary crystallographic information

### Experimental

The Schiff base ligand was prepared by the condensation of 3-hydroxy-2-naphthoiyl hydrazide with 2-hydroxyacetophenone. The organotin compound was prepared by heating the Schiff base (0.64 g, 2 mmol) and dicyclohexyltin oxide (0.6 g, 2mmol) in toluene for 3 h. After filtering the solution, yellow crystals were obtained upon evaporation of the filtrate.

### Refinement

H atoms were placed at calculated positions (C–H = 0.93–0.98 Å and O–H = 0.82 Å) and were treated as riding on their parent atoms, with *U*(H) set to 1.2–1.5 times *U*_eq_(C,O).

### Crystal data

**Table:**

[Sn(C_6_H_11_)_2_(C_19_H_14_N_2_O_3_)]	*F*(000) = 2480
*M**_r_* = 603.31	*D*_x_ = 1.428 Mg m^−^^3^
Monoclinic, *C*2/*c*	Mo *K*α radiation, λ = 0.71073 Å
Hall symbol: -C 2yc	Cell parameters from 4113 reflections
*a* = 30.2358 (4) Å	θ = 2.6–20.3°
*b* = 7.7030 (1) Å	µ = 0.95 mm^−^^1^
*c* = 25.8528 (4) Å	*T* = 293 K
β = 111.249 (2)°	Block, yellow
*V* = 5611.92 (16) Å^3^	0.30 × 0.10 × 0.10 mm
*Z* = 8	

### Data collection

**Table:**

Bruker SMART APEX diffractometer	6424 independent reflections
Radiation source: fine-focus sealed tube	3942 reflections with *I* > 2σ(*I*)
graphite	*R*_int_ = 0.047
ω scans	θ_max_ = 27.5°, θ_min_ = 1.7°
Absorption correction: multi-scan (*SADABS*; Sheldrick, 1996)	*h* = −39→39
*T*_min_ = 0.765, *T*_max_ = 0.912	*k* = −10→10
26097 measured reflections	*l* = −33→33

### Refinement

**Table:**

Refinement on *F*^2^	Primary atom site location: structure-invariant direct methods
Least-squares matrix: full	Secondary atom site location: difference Fourier map
*R*[*F*^2^ > 2σ(*F*^2^)] = 0.043	Hydrogen site location: inferred from neighbouring sites
*wR*(*F*^2^) = 0.111	H-atom parameters constrained
*S* = 1.00	*w* = 1/[σ^2^(*F*_o_^2^) + (0.047*P*)^2^ + 3.9403*P*] where *P* = (*F*_o_^2^ + 2*F*_c_^2^)/3
6424 reflections	(Δ/σ)_max_ = 0.001
336 parameters	Δρ_max_ = 0.52 e Å^−^^3^
0 restraints	Δρ_min_ = −0.43 e Å^−^^3^

### Fractional atomic coordinates and isotropic or equivalent isotropic displacement parameters (Å^2^)

**Table:**

	*x*	*y*	*z*	*U*_iso_*/*U*_eq_	
Sn1	0.607817 (9)	0.32667 (4)	0.446538 (11)	0.06297 (12)	
O1	0.59313 (11)	0.1555 (4)	0.38108 (11)	0.0778 (8)	
O2	0.65161 (9)	0.5072 (4)	0.50781 (11)	0.0713 (7)	
O3	0.79451 (10)	0.5522 (5)	0.52370 (13)	0.0853 (9)	
H3	0.7728	0.5030	0.4994	0.128*	
N1	0.67212 (11)	0.3711 (4)	0.42833 (13)	0.0574 (8)	
N2	0.70928 (10)	0.4444 (4)	0.47247 (13)	0.0589 (8)	
C1	0.61233 (16)	0.1276 (6)	0.50638 (17)	0.0735 (11)	
H1	0.6394	0.0557	0.5075	0.088*	
C2	0.6237 (3)	0.1871 (7)	0.5631 (2)	0.134 (3)	
H2A	0.5998	0.2696	0.5641	0.161*	
H2B	0.6540	0.2470	0.5753	0.161*	
C3	0.6262 (3)	0.0403 (9)	0.6031 (2)	0.159 (3)	
H3A	0.6546	−0.0276	0.6085	0.191*	
H3B	0.6289	0.0891	0.6387	0.191*	
C4	0.5843 (3)	−0.0760 (9)	0.5836 (2)	0.137 (3)	
H4A	0.5899	−0.1750	0.6083	0.165*	
H4B	0.5569	−0.0141	0.5852	0.165*	
C5	0.5741 (2)	−0.1371 (6)	0.5276 (2)	0.1075 (18)	
H5A	0.5446	−0.2017	0.5155	0.129*	
H5B	0.5991	−0.2161	0.5274	0.129*	
C6	0.57024 (18)	0.0080 (6)	0.48669 (18)	0.0939 (15)	
H6A	0.5677	−0.0415	0.4512	0.113*	
H6B	0.5416	0.0742	0.4812	0.113*	
C7	0.55119 (15)	0.5063 (6)	0.41036 (17)	0.0755 (11)	
H7	0.5541	0.5965	0.4381	0.091*	
C8	0.50407 (19)	0.4219 (9)	0.3978 (4)	0.163 (3)	
H8A	0.5022	0.3764	0.4319	0.196*	
H8B	0.5013	0.3249	0.3728	0.196*	
C9	0.4627 (2)	0.5467 (11)	0.3712 (4)	0.189 (4)	
H9A	0.4331	0.4828	0.3607	0.227*	
H9B	0.4625	0.6339	0.3982	0.227*	
C10	0.4659 (2)	0.6324 (9)	0.3222 (3)	0.154 (3)	
H10A	0.4406	0.7171	0.3085	0.185*	
H10B	0.4616	0.5469	0.2933	0.185*	
C11	0.5115 (3)	0.7192 (8)	0.3344 (3)	0.138 (2)	
H11A	0.5141	0.8146	0.3598	0.166*	
H11B	0.5130	0.7670	0.3004	0.166*	
C12	0.5529 (2)	0.5950 (8)	0.3600 (3)	0.124 (2)	
H12A	0.5526	0.5082	0.3327	0.149*	
H12B	0.5824	0.6591	0.3698	0.149*	
C13	0.60322 (16)	0.1758 (5)	0.33577 (16)	0.0663 (10)	
C14	0.57067 (18)	0.1090 (6)	0.28635 (18)	0.0838 (13)	
H14	0.5428	0.0581	0.2863	0.101*	
C15	0.5795 (2)	0.1180 (7)	0.2377 (2)	0.1016 (17)	
H15	0.5575	0.0737	0.2051	0.122*	
C16	0.6205 (2)	0.1918 (7)	0.2375 (2)	0.1091 (19)	
H16	0.6265	0.1971	0.2047	0.131*	
C17	0.65253 (19)	0.2574 (6)	0.28514 (19)	0.0844 (14)	
H17	0.6802	0.3074	0.2842	0.101*	
C18	0.64529 (15)	0.2524 (5)	0.33554 (16)	0.0633 (10)	
C19	0.68174 (14)	0.3258 (4)	0.38474 (16)	0.0592 (9)	
C20	0.73134 (15)	0.3514 (5)	0.38609 (19)	0.0770 (12)	
H20A	0.7536	0.3110	0.4209	0.115*	
H20B	0.7367	0.4725	0.3818	0.115*	
H20C	0.7355	0.2870	0.3564	0.115*	
C21	0.69499 (13)	0.5079 (5)	0.51059 (16)	0.0572 (9)	
C22	0.73168 (13)	0.5798 (5)	0.56094 (15)	0.0555 (9)	
C23	0.78029 (14)	0.5961 (5)	0.56581 (17)	0.0626 (10)	
C24	0.81286 (15)	0.6568 (5)	0.61388 (19)	0.0742 (12)	
H24	0.8443	0.6672	0.6166	0.089*	
C25	0.80107 (15)	0.7043 (5)	0.65944 (18)	0.0681 (11)	
C26	0.83469 (19)	0.7662 (7)	0.7099 (2)	0.0911 (15)	
H26	0.8664	0.7745	0.7139	0.109*	
C27	0.8213 (2)	0.8136 (6)	0.7525 (2)	0.1030 (18)	
H27	0.8439	0.8551	0.7852	0.124*	
C28	0.7746 (2)	0.8012 (6)	0.7481 (2)	0.0982 (17)	
H28	0.7659	0.8346	0.7776	0.118*	
C29	0.74128 (18)	0.7402 (6)	0.70071 (19)	0.0833 (13)	
H29	0.7099	0.7313	0.6983	0.100*	
C30	0.75344 (15)	0.6904 (5)	0.65528 (17)	0.0658 (10)	
C31	0.71993 (14)	0.6266 (5)	0.60520 (16)	0.0634 (10)	
H31	0.6885	0.6159	0.6023	0.076*	

### Atomic displacement parameters (Å^2^)

**Table:**

	*U*^11^	*U*^22^	*U*^33^	*U*^12^	*U*^13^	*U*^23^
Sn1	0.05673 (17)	0.0795 (2)	0.05493 (17)	−0.00534 (14)	0.02301 (13)	−0.00016 (14)
O1	0.083 (2)	0.095 (2)	0.0615 (17)	−0.0239 (16)	0.0339 (15)	−0.0086 (15)
O2	0.0561 (16)	0.086 (2)	0.0739 (17)	−0.0072 (14)	0.0266 (14)	−0.0162 (15)
O3	0.0598 (18)	0.116 (3)	0.085 (2)	0.0003 (17)	0.0322 (17)	−0.0030 (18)
N1	0.0606 (19)	0.0569 (19)	0.0572 (18)	0.0028 (14)	0.0243 (16)	0.0065 (14)
N2	0.0561 (19)	0.061 (2)	0.0605 (19)	0.0022 (15)	0.0226 (16)	0.0075 (15)
C1	0.073 (3)	0.093 (3)	0.061 (2)	−0.004 (2)	0.032 (2)	0.004 (2)
C2	0.196 (7)	0.126 (5)	0.064 (3)	−0.078 (4)	0.028 (4)	0.000 (3)
C3	0.223 (8)	0.165 (6)	0.058 (3)	−0.083 (6)	0.014 (4)	0.016 (4)
C4	0.201 (7)	0.139 (5)	0.073 (4)	−0.058 (5)	0.052 (4)	0.008 (3)
C5	0.151 (5)	0.095 (4)	0.084 (4)	−0.030 (3)	0.050 (4)	0.002 (3)
C6	0.108 (4)	0.102 (4)	0.065 (3)	−0.033 (3)	0.024 (3)	−0.002 (3)
C7	0.073 (3)	0.083 (3)	0.065 (3)	0.006 (2)	0.019 (2)	0.000 (2)
C8	0.069 (4)	0.155 (6)	0.271 (9)	0.025 (4)	0.068 (5)	0.112 (6)
C9	0.080 (4)	0.181 (8)	0.299 (11)	0.028 (5)	0.061 (6)	0.110 (8)
C10	0.105 (5)	0.104 (5)	0.185 (8)	−0.004 (4)	−0.030 (5)	0.023 (5)
C11	0.130 (6)	0.107 (5)	0.153 (6)	0.019 (4)	0.021 (5)	0.051 (4)
C12	0.104 (4)	0.117 (4)	0.150 (6)	0.003 (4)	0.043 (4)	0.049 (4)
C13	0.083 (3)	0.064 (2)	0.056 (2)	0.000 (2)	0.030 (2)	0.0012 (19)
C14	0.101 (4)	0.085 (3)	0.065 (3)	−0.023 (3)	0.030 (3)	−0.013 (2)
C15	0.135 (5)	0.103 (4)	0.063 (3)	−0.021 (3)	0.031 (3)	−0.017 (3)
C16	0.159 (6)	0.115 (4)	0.069 (3)	−0.038 (4)	0.060 (4)	−0.019 (3)
C17	0.116 (4)	0.081 (3)	0.071 (3)	−0.016 (3)	0.052 (3)	−0.006 (2)
C18	0.085 (3)	0.053 (2)	0.061 (2)	0.005 (2)	0.036 (2)	0.0049 (18)
C19	0.072 (2)	0.050 (2)	0.063 (2)	0.0068 (19)	0.033 (2)	0.0122 (18)
C20	0.078 (3)	0.080 (3)	0.087 (3)	0.009 (2)	0.047 (3)	0.010 (2)
C21	0.057 (2)	0.052 (2)	0.063 (2)	0.0032 (18)	0.0227 (19)	0.0066 (18)
C22	0.053 (2)	0.052 (2)	0.061 (2)	0.0032 (17)	0.0207 (19)	0.0114 (17)
C23	0.057 (2)	0.061 (2)	0.069 (3)	0.0050 (18)	0.022 (2)	0.012 (2)
C24	0.054 (2)	0.073 (3)	0.086 (3)	0.006 (2)	0.014 (2)	0.012 (2)
C25	0.071 (3)	0.053 (2)	0.068 (3)	0.0006 (19)	0.009 (2)	0.0097 (19)
C26	0.078 (3)	0.090 (3)	0.085 (4)	0.000 (3)	0.004 (3)	0.005 (3)
C27	0.116 (5)	0.089 (4)	0.076 (3)	−0.015 (3)	0.002 (3)	0.000 (3)
C28	0.130 (5)	0.093 (4)	0.068 (3)	−0.022 (3)	0.031 (3)	−0.013 (3)
C29	0.094 (4)	0.083 (3)	0.076 (3)	−0.015 (3)	0.035 (3)	−0.006 (2)
C30	0.076 (3)	0.052 (2)	0.064 (2)	−0.0053 (19)	0.018 (2)	0.0067 (19)
C31	0.061 (2)	0.059 (2)	0.070 (3)	−0.0011 (18)	0.024 (2)	0.0052 (19)

### Geometric parameters (Å, °)

**Table:**

Sn1—O1	2.063 (3)	C10—H10B	0.97
Sn1—C7	2.136 (4)	C11—C12	1.523 (8)
Sn1—C1	2.147 (4)	C11—H11A	0.97
Sn1—O2	2.162 (3)	C11—H11B	0.97
Sn1—N1	2.187 (3)	C12—H12A	0.97
O1—C13	1.322 (5)	C12—H12B	0.97
O2—C21	1.287 (4)	C13—C18	1.404 (6)
O3—C23	1.350 (5)	C13—C14	1.398 (6)
O3—H3	0.82	C14—C15	1.378 (6)
N1—C19	1.309 (5)	C14—H14	0.93
N1—N2	1.398 (4)	C15—C16	1.367 (8)
N2—C21	1.306 (5)	C15—H15	0.93
C1—C2	1.455 (6)	C16—C17	1.359 (7)
C1—C6	1.502 (6)	C16—H16	0.93
C1—H1	0.98	C17—C18	1.398 (6)
C2—C3	1.515 (7)	C17—H17	0.93
C2—H2A	0.97	C18—C19	1.461 (6)
C2—H2B	0.97	C19—C20	1.501 (5)
C3—C4	1.483 (8)	C20—H20A	0.96
C3—H3A	0.97	C20—H20B	0.96
C3—H3B	0.97	C20—H20C	0.96
C4—C5	1.447 (7)	C21—C22	1.478 (5)
C4—H4A	0.97	C22—C31	1.364 (5)
C4—H4B	0.97	C22—C23	1.434 (5)
C5—C6	1.514 (6)	C23—C24	1.358 (6)
C5—H5A	0.97	C24—C25	1.397 (6)
C5—H5B	0.97	C24—H24	0.93
C6—H6A	0.97	C25—C30	1.409 (6)
C6—H6B	0.97	C25—C26	1.415 (6)
C7—C12	1.487 (7)	C26—C27	1.354 (7)
C7—C8	1.491 (7)	C26—H26	0.93
C7—H7	0.98	C27—C28	1.377 (8)
C8—C9	1.529 (8)	C27—H27	0.93
C8—H8A	0.97	C28—C29	1.357 (6)
C8—H8B	0.97	C28—H28	0.93
C9—C10	1.463 (10)	C29—C30	1.405 (6)
C9—H9A	0.97	C29—H29	0.93
C9—H9B	0.97	C30—C31	1.412 (5)
C10—C11	1.460 (9)	C31—H31	0.93
C10—H10A	0.97		
			
O1—Sn1—C7	98.91 (14)	C11—C10—H10B	109.2
O1—Sn1—C1	94.19 (15)	H10A—C10—H10B	107.9
C7—Sn1—C1	127.59 (17)	C10—C11—C12	111.6 (5)
O1—Sn1—O2	154.78 (11)	C10—C11—H11A	109.3
C7—Sn1—O2	94.88 (14)	C12—C11—H11A	109.3
C1—Sn1—O2	93.98 (14)	C10—C11—H11B	109.3
O1—Sn1—N1	82.38 (11)	C12—C11—H11B	109.3
C7—Sn1—N1	116.00 (14)	H11A—C11—H11B	108.0
C1—Sn1—N1	115.95 (14)	C7—C12—C11	112.7 (5)
O2—Sn1—N1	72.59 (11)	C7—C12—H12A	109.1
C13—O1—Sn1	126.9 (2)	C11—C12—H12A	109.1
C21—O2—Sn1	112.6 (2)	C7—C12—H12B	109.1
C23—O3—H3	109.5	C11—C12—H12B	109.1
C19—N1—N2	116.5 (3)	H12A—C12—H12B	107.8
C19—N1—Sn1	129.5 (3)	O1—C13—C18	123.6 (4)
N2—N1—Sn1	113.7 (2)	O1—C13—C14	117.0 (4)
C21—N2—N1	112.3 (3)	C18—C13—C14	119.3 (4)
C2—C1—C6	113.3 (4)	C15—C14—C13	120.7 (5)
C2—C1—Sn1	115.6 (3)	C15—C14—H14	119.7
C6—C1—Sn1	111.6 (3)	C13—C14—H14	119.7
C2—C1—H1	105.0	C16—C15—C14	120.1 (5)
C6—C1—H1	105.0	C16—C15—H15	119.9
Sn1—C1—H1	105.0	C14—C15—H15	119.9
C1—C2—C3	112.8 (5)	C17—C16—C15	120.0 (5)
C1—C2—H2A	109.0	C17—C16—H16	120.0
C3—C2—H2A	109.0	C15—C16—H16	120.0
C1—C2—H2B	109.0	C16—C17—C18	122.3 (5)
C3—C2—H2B	109.0	C16—C17—H17	118.8
H2A—C2—H2B	107.8	C18—C17—H17	118.8
C4—C3—C2	113.2 (5)	C17—C18—C13	117.6 (4)
C4—C3—H3A	108.9	C17—C18—C19	118.5 (4)
C2—C3—H3A	108.9	C13—C18—C19	123.9 (3)
C4—C3—H3B	108.9	N1—C19—C18	121.3 (4)
C2—C3—H3B	108.9	N1—C19—C20	118.5 (4)
H3A—C3—H3B	107.8	C18—C19—C20	120.2 (4)
C5—C4—C3	112.5 (5)	C19—C20—H20A	109.5
C5—C4—H4A	109.1	C19—C20—H20B	109.5
C3—C4—H4A	109.1	H20A—C20—H20B	109.5
C5—C4—H4B	109.1	C19—C20—H20C	109.5
C3—C4—H4B	109.1	H20A—C20—H20C	109.5
H4A—C4—H4B	107.8	H20B—C20—H20C	109.5
C4—C5—C6	113.2 (5)	O2—C21—N2	124.4 (4)
C4—C5—H5A	108.9	O2—C21—C22	118.4 (3)
C6—C5—H5A	108.9	N2—C21—C22	117.2 (3)
C4—C5—H5B	108.9	C31—C22—C23	118.3 (4)
C6—C5—H5B	108.9	C31—C22—C21	119.8 (3)
H5A—C5—H5B	107.8	C23—C22—C21	121.9 (4)
C1—C6—C5	112.0 (4)	O3—C23—C24	119.1 (4)
C1—C6—H6A	109.2	O3—C23—C22	121.5 (4)
C5—C6—H6A	109.2	C24—C23—C22	119.4 (4)
C1—C6—H6B	109.2	C23—C24—C25	122.6 (4)
C5—C6—H6B	109.2	C23—C24—H24	118.7
H6A—C6—H6B	107.9	C25—C24—H24	118.7
C12—C7—C8	110.0 (5)	C24—C25—C30	118.8 (4)
C12—C7—Sn1	113.8 (3)	C24—C25—C26	123.2 (5)
C8—C7—Sn1	111.3 (3)	C30—C25—C26	118.0 (5)
C12—C7—H7	107.1	C27—C26—C25	120.9 (5)
C8—C7—H7	107.1	C27—C26—H26	119.5
Sn1—C7—H7	107.1	C25—C26—H26	119.5
C7—C8—C9	112.7 (5)	C26—C27—C28	120.9 (5)
C7—C8—H8A	109.1	C26—C27—H27	119.5
C9—C8—H8A	109.1	C28—C27—H27	119.5
C7—C8—H8B	109.1	C29—C28—C27	120.1 (5)
C9—C8—H8B	109.1	C29—C28—H28	119.9
H8A—C8—H8B	107.8	C27—C28—H28	119.9
C10—C9—C8	111.8 (7)	C28—C29—C30	121.0 (5)
C10—C9—H9A	109.3	C28—C29—H29	119.5
C8—C9—H9A	109.3	C30—C29—H29	119.5
C10—C9—H9B	109.3	C25—C30—C29	119.0 (4)
C8—C9—H9B	109.3	C25—C30—C31	118.1 (4)
H9A—C9—H9B	107.9	C29—C30—C31	122.9 (4)
C9—C10—C11	112.0 (6)	C22—C31—C30	122.9 (4)
C9—C10—H10A	109.2	C22—C31—H31	118.6
C11—C10—H10A	109.2	C30—C31—H31	118.6
C9—C10—H10B	109.2		
			
C7—Sn1—O1—C13	78.9 (4)	Sn1—O1—C13—C14	−145.0 (3)
C1—Sn1—O1—C13	−152.0 (4)	O1—C13—C14—C15	−177.2 (4)
O2—Sn1—O1—C13	−43.4 (5)	C18—C13—C14—C15	−0.1 (7)
N1—Sn1—O1—C13	−36.4 (3)	C13—C14—C15—C16	0.4 (8)
O1—Sn1—O2—C21	−9.9 (4)	C14—C15—C16—C17	−0.4 (9)
C7—Sn1—O2—C21	−133.0 (3)	C15—C16—C17—C18	0.1 (9)
C1—Sn1—O2—C21	98.7 (3)	C16—C17—C18—C13	0.2 (7)
N1—Sn1—O2—C21	−17.3 (2)	C16—C17—C18—C19	179.8 (5)
O1—Sn1—N1—C19	13.7 (3)	O1—C13—C18—C17	176.7 (4)
C7—Sn1—N1—C19	−82.5 (3)	C14—C13—C18—C17	−0.2 (6)
C1—Sn1—N1—C19	104.7 (3)	O1—C13—C18—C19	−3.0 (6)
O2—Sn1—N1—C19	−169.5 (3)	C14—C13—C18—C19	−179.9 (4)
O1—Sn1—N1—N2	−159.7 (2)	N2—N1—C19—C18	−178.3 (3)
C7—Sn1—N1—N2	104.0 (2)	Sn1—N1—C19—C18	8.4 (5)
C1—Sn1—N1—N2	−68.8 (3)	N2—N1—C19—C20	1.5 (5)
O2—Sn1—N1—N2	17.1 (2)	Sn1—N1—C19—C20	−171.8 (2)
C19—N1—N2—C21	170.9 (3)	C17—C18—C19—N1	160.1 (4)
Sn1—N1—N2—C21	−14.8 (3)	C13—C18—C19—N1	−20.3 (6)
O1—Sn1—C1—C2	178.9 (5)	C17—C18—C19—C20	−19.7 (6)
C7—Sn1—C1—C2	−76.5 (5)	C13—C18—C19—C20	159.9 (4)
O2—Sn1—C1—C2	22.8 (5)	Sn1—O2—C21—N2	16.8 (5)
N1—Sn1—C1—C2	95.4 (5)	Sn1—O2—C21—C22	−161.0 (2)
O1—Sn1—C1—C6	−49.6 (3)	N1—N2—C21—O2	−1.3 (5)
C7—Sn1—C1—C6	55.0 (4)	N1—N2—C21—C22	176.5 (3)
O2—Sn1—C1—C6	154.3 (3)	O2—C21—C22—C31	6.7 (5)
N1—Sn1—C1—C6	−133.2 (3)	N2—C21—C22—C31	−171.3 (3)
C6—C1—C2—C3	49.2 (8)	O2—C21—C22—C23	−175.8 (3)
Sn1—C1—C2—C3	179.9 (5)	N2—C21—C22—C23	6.3 (5)
C1—C2—C3—C4	−49.9 (10)	C31—C22—C23—O3	180.0 (4)
C2—C3—C4—C5	51.4 (9)	C21—C22—C23—O3	2.4 (6)
C3—C4—C5—C6	−52.4 (9)	C31—C22—C23—C24	0.1 (5)
C2—C1—C6—C5	−49.8 (6)	C21—C22—C23—C24	−177.5 (4)
Sn1—C1—C6—C5	177.6 (4)	O3—C23—C24—C25	−179.7 (4)
C4—C5—C6—C1	51.4 (7)	C22—C23—C24—C25	0.2 (6)
O1—Sn1—C7—C12	−62.2 (4)	C23—C24—C25—C30	−0.7 (6)
C1—Sn1—C7—C12	−164.5 (4)	C23—C24—C25—C26	179.4 (4)
O2—Sn1—C7—C12	96.6 (4)	C24—C25—C26—C27	178.8 (4)
N1—Sn1—C7—C12	23.6 (4)	C30—C25—C26—C27	−1.2 (7)
O1—Sn1—C7—C8	62.9 (5)	C25—C26—C27—C28	0.7 (8)
C1—Sn1—C7—C8	−39.4 (5)	C26—C27—C28—C29	0.2 (8)
O2—Sn1—C7—C8	−138.3 (4)	C27—C28—C29—C30	−0.6 (8)
N1—Sn1—C7—C8	148.7 (4)	C24—C25—C30—C29	−179.1 (4)
C12—C7—C8—C9	−52.2 (9)	C26—C25—C30—C29	0.8 (6)
Sn1—C7—C8—C9	−179.4 (6)	C24—C25—C30—C31	0.9 (5)
C7—C8—C9—C10	53.5 (10)	C26—C25—C30—C31	−179.2 (4)
C8—C9—C10—C11	−54.2 (10)	C28—C29—C30—C25	0.1 (7)
C9—C10—C11—C12	54.9 (10)	C28—C29—C30—C31	−179.9 (4)
C8—C7—C12—C11	52.9 (7)	C23—C22—C31—C30	0.2 (6)
Sn1—C7—C12—C11	178.7 (4)	C21—C22—C31—C30	177.8 (3)
C10—C11—C12—C7	−54.9 (9)	C25—C30—C31—C22	−0.6 (6)
Sn1—O1—C13—C18	38.1 (6)	C29—C30—C31—C22	179.4 (4)

### Hydrogen-bond geometry (Å, °)

**Table:**

*D*—H···*A*	*D*—H	H···*A*	*D*···*A*	*D*—H···*A*
O3—H3···N2	0.82	1.85	2.571 (4)	147
